# The Patient's Point of View: Characterizing Patient-Level Factors Associated with Perceptions of Health Care

**DOI:** 10.1089/heq.2021.0062

**Published:** 2021-06-25

**Authors:** Maya J. Torain, Gary G. Bennett, Roland A. Matsouaka, Maren K. Olsen, Hongqiu Yang, Jamiyla H. Bolton, Kimberly S. Johnson, Laura P. Svetkey

**Affiliations:** ^1^ Duke Center for Research to Advance Healthcare Equity, Durham, North Carolina, USA.; ^2^ Duke University School of Medicine, Durham, North Carolina, USA.; ^3^ Department of Psychology and Neuroscience, Duke Global Digital Health Science Center, Duke Global Health Institute, Duke University, Durham, North Carolina, USA.; ^4^ Department of Psychology and Neuroscience, Duke University, Durham, North Carolina, USA.; ^5^ Department of Biostatistics and Bioinformatics, Duke University, Durham, North Carolina, USA.; ^6^ Duke Clinical Research Institute, Durham, North Carolina, USA.; ^7^ Durham Center of Innovation to Accelerate Discovery and Practice Transformation, Durham Veterans Affairs Health Care System, Durham, North Carolina, USA.; ^8^ Geriatrics Research Education and Clinical Center, Durham Veterans Affairs Administration, Durham, North Carolina, USA.; ^9^ Department of Medicine, Duke University Medical Center, Durham, North Carolina, USA.

**Keywords:** patient perception, communication, health disparities, patient-centered care

## Abstract

**Purpose:** We explored the association between perception of care, as measured by the Interpersonal Processes of Care (IPC) survey, and patient-level factors, including (1) Trust in physicians; (2) Perceived empathy; (3) Stereotype threat; (4) Perceived everyday discrimination; and (5) Self-Reported Health.

**Methods:** Fifty participants from diverse racial backgrounds and education levels were surveyed. We examined the associations between the five patient-level factors and each subdomain of the IPC using multiple linear regression. We added a race interaction term to assess whether associations between IPC subdomains and predictors differed by race. We tested for correlation among factors found to be significantly associated with the IPC.

**Results:** In adjusted analyses, trust in the physician, perceived empathy from the provider, and perceived everyday discrimination were significantly associated with most subdomains of the IPC. There was no significant race interaction.

**Conclusion:** This exploratory study suggests that empathy, trust, and perceived everyday discrimination are significantly linked to patient perception of quality care, which are linked to clinical outcomes. Results present modifiable factors that may potentially improve patient care.

**Practice Implications:** Increased efforts to improve clinician communication of empathy and general communication skill may have a positive effect on quality of care.

## Introduction

Racial and ethnic health care disparities are widely described in the literature and are associated with increased morbidity and mortality, and lower quality patient care experience. These disparities persist after controlling for differences in socioeconomic status and access to care, suggesting that the etiology of health care disparities is multifactorial, with patient-, provider-, hospital-, and system-level factors contributing.^1,2^ Patient-level factors include both fixed characteristics like sociodemographic variables and those that may be modifiable based on patient-physician-system interactions.^1^

Attending to patient-level factors reflects patient-centered care. In 2001, the Institute of Medicine described patient centeredness as one of the six core needs of quality health care and promoted specific aims for improvement.^3^ Patient centeredness is defined as “providing care that is respectful of and responsive to individual patient preferences, needs, and values, and ensuring that patient values guide all clinical decisions” (IOM, p. 40).^3^ Central to the delivery of patient-centered care is understanding the patient perspective and factors that shape it.^4^ Patient perceptions of their interaction with physicians, hospitals, and health systems are frequently assessed in patient satisfaction surveys and widely used as an important indicator in evaluation of care quality.^5,6^

Compared to whites, racial and ethnic minorities have fewer positive perceptions of the care experience, which may contribute to disparities.^7–9^ Patient perception of care has been significantly associated with health outcomes.^10,11^ Studies have shown association between positive perceptions of care and improved health outcomes and patient adherence in patients with HIV, depression, and diabetes.^12–15^ In a study by Stewart, patients' perceptions of patient centeredness were associated with improved health status and efficiency of care (reduced diagnostic tests and referrals).^11^ In contrast, there was no significant association between expert rating of patient centeredness in audiotaped clinical encounters and outcomes, highlighting the relative importance of the perception of patients themselves.^11^

These findings suggest a potential mechanism for improving health outcomes and reducing disparities through improvement of patient perceptions of care. Patient perceptions of the quality of their interaction with providers may affect other factors associated with health care outcomes, including physician trust, perceived discrimination, perceived empathy, and the experience of stereotype threat. However, there are few studies that characterize the factors and values that shape this perception. The objective of this study was to determine the association between perception of care, as measured by the Interpersonal Processes of Care (IPC) survey,^16^ and five patient-level factors that may be intermediate measures on the path to health outcomes through impact on patient perception: trust in physicians, perceived empathy, perceived everyday discrimination, stereotype threat, and self-reported health. This work fills a gap in the literature through an exploratory analysis of patient-level factors that may impact perception of care delivery. Further understanding of patient perception may aid in the mission to deliver equitable, quality patient-centered care.

## Methods

### Study design

We conducted this study concurrently with a research project in the Duke Center for Research to Advance Healthcare Equity (REACH Equity; NIH: grant No. U54MD012530). The purpose of the overall project is to develop and pilot test an implicit bias training intervention for providers. The first phase of this project involved a series of focus groups to identify patient experiences in the clinical encounter that might reflect or be identified by patients as influenced by implicit bias. We conducted six focus groups with participants from diverse racial, ethnic, and socioeconomic backgrounds. Immediately following the focus group, participants completed a series of surveys detailed below. The analysis reported in this study is based on the survey data. This study was approved by the Duke University Medical Center Institutional Review Board. We obtained online informed consent from all participants.

### Study population

All participants from the focus groups (*N*=50) are included in this analysis. Recruitment took place at a large academic center, through local flyers and online platforms. The inclusion criteria were as follows: (1) 18 years of age or older, (2) English language proficient, and (3) having at least two nonurgent ambulatory care visits within the last 12 months.

### Materials/survey

Demographic characteristics, obtained by self-report, included age, race, ethnicity, gender at birth, gender described, education, marital status, employment, insurance, and income.

### IPC (dependent variables)

We administered the IPC survey^16^ to assess perceptions of quality of care. The IPC is a 29-item instrument designed to assess perceived quality of care based upon social-psychological components of the patient-physician interaction. It is divided into three key domains (communication, decision making, and interpersonal style) with seven subdomains (Table 1). The IPC is scored by subdomains with directionality of score varying by subdomain. Each item is assessed using a 5-point Likert scale. Items were asked in reference to patient ambulatory care experiences over the past 12 months. The IPC subdomain scores served as dependent variables. As there is no validated composite score for the IPC, we analyzed each subdomain as an independent outcome.

**Table 1.** Survey Characteristics and Results

	Potential range	Median (25th, 75th)
IPC	1–5	
IPC Domain 1: Hurried communication (−)		2.6 (2.2, 3.0)
IPC Domain 2: Elicited concerns, responded (+)		3.8 (3.0, 4.3)
IPC Domain 3: Explained results, medications (+)		3.5 (2.8, 4.0)
IPC Domain 4: Patient-centered decision making (+)		3 (2.0, 3.9)
IPC Domain 5: Compassionate, respectful (+)		3.6 (3.0, 4.3)
IPC Domain 6: Discriminated (−)		2 (1.3, 3.0)
IPC Domain 7: Disrespectful office staff (−)		2 (1.0, 2.9)
Trust in physician (+)	5–25	18 (15.0, 19.0)
CARE measure (+)	10–50	37 (28.0, 50.0)
Stereotype vulnerability (−)	1–5	2.5 (1.5, 3.0)
Everyday discrimination (−)	0–45	12.5 (7.5, 15.0)
Self-rated health (+)	1–5	4 (3.0, 5.0)

(−) Indicates that a higher score=worse outcome; (+) indicates that a higher score=better outcome.

CARE, consultation and relational empathy; IPC, Interpersonal Processes of Care.

### Independent variable measures

We assessed the patient-level factors of interest with five validated surveys: (1) Trust in Physician survey (5-item)^17^; (2) Consultation and relational empathy (CARE) measure (10-item)^18^; (3) Stereotype Vulnerability Scale (SVS-4; 4-item)^19,20^; (4) Everyday Discrimination Scale (9-item)^21,22^; and (5) Self-reported health.^23,24^ We chose these measures because they vary by race and are associated with health outcomes.^25–29^ These measures served as independent (predictor) variables (Supplementary Appendix SA1).

### Trust

The Trust in Physicians survey is a 5-item instrument used to assess patient trust in individual medical providers. Each item is assessed using a 5-point Likert scale (1–5), and responses are summed. Total scores range from 5 to 25 with higher values indicating more trust.^17^

### Empathy

The CARE measure is a 10-item instrument developed to assess perceptions of relational empathy in clinical settings.^18^ Each item is assessed using a 5-point Likert scale, and responses are summed. Total scores range from 10 to 50 with higher values indicating greater perceptions of empathy displayed by physicians.^18^

### Stereotype vulnerability

Stereotype threat is “the tendency to expect, perceive, and be influenced by negative stereotypes about one's social category.”^30^ We assessed stereotype threat using the revised version of the SVS. Each of four items is assessed using a 5-point Likert scale, and responses are averaged to calculate the composite score. Total scores range from 1 to 5 with higher scores indicating greater vulnerability.^19,31^

### Perceived discrimination

The Everyday Discrimination Scale is a 9-item instrument used to assess frequency of perceived discrimination in day-to-day life. Total scores range from 0 to 45 with higher scores indicating greater perceived discrimination.^21,22,32^

### Health

Self-reported health was measured using a single-item indicator of general health status. Responses were assessed using a 5-point Likert scale ranging from 1 (Poor) to 5 (Excellent).^23,24^ Similar to its use elsewhere,^27,32–34^ self-reported health was analyzed by combining scores 1–3 versus 4–5, which divided the study population approximately in half.

### Analysis

Descriptive statistics were tabulated for demographic data, the IPC subdomains, and patient-level factors. We examined the associations between each subdomain of the IPC (dependent variable) and the five patient-level factors (independent variables) by multiple linear regression. We created one regression model for each of the patient-level factors (trust, perceived empathy, stereotype threat, perceived discrimination, and health). Models were adjusted for age, sex, race, ethnicity, and education. Because of small sample size, we conducted an exploratory approach to analysis, without stratification by patient characteristic or correction for multiple comparisons. To make regression coefficients potentially more reflective of meaningful increments in IPC scores, we scaled predictor variables so that coefficients reflect effects of a 1-, 5- or 10-unit change in the independent variable.^35^ We added a race interaction term to each model to assess whether associations between IPC subdomains and predictors differed by race. Race was grouped into white versus non-white due to small sample size. There were insignificant numbers of Latinx participants to assess for interaction by ethnicity. In addition, we calculated correlations among those patient-level factors that were found to be significantly associated (*p*<0.05) with more than one IPC subdomain. This was done to determine if the factors were surrogates for each other, All analyses were completed using SAS University Edition.

## Results

### Sample characteristics

As noted in Table 2, of 50 participants, 80% were female with a mean age of 42.4 years (range 21–81). Participants were 40% black, 52% white, and 22% identified as Hispanic/Latinx ethnicity. For each IPC subdomain and patient-level factor survey, there was a 92–98% participant completion rate.

**Table 2.** Participant Characteristics

	Total, *N*=50
Age (years), mean (SD)	42.4 (15.9)
Nonambulatory visits in past 12 months	
No. of visits, median (25th, 75th)	5.5 (3.0, 10.0)
Sex, *N* (%)	
Female	40 (80)
Race, *N* (%)	
Black	20 (40)
White	26 (52)
Other	4 (8)
Ethnicity, *N* (%)	
Hispanic/Latinx	11 (22)
Education, *N* (%)	
Bachelor's degree or higher	31 (62)

SD, standard deviation.

### Association between patient-level factors and IPC

In adjusted analyses, trust in the physician, perceived empathy from the provider, and perceived everyday discrimination were significantly associated with most subdomains of the IPC (Table 3). Specifically, greater perceived empathy in a clinical setting was associated with the perception that doctors have adequately elicited concerns, explained results and medications, engaged in patient-centered decision making, and demonstrated compassionate, respectful care (all *p*<0.01). Higher levels of trust in physicians were associated with the perception that doctors have adequately elicited concerns (*p*=0.041), explained results and medications (*p*=0.0004), engaged in patient-centered decision making (*p*=0.0001), and demonstrated compassionate, respectful care (*p*=0.0019). Greater perceived everyday discrimination was associated with the perception of hurried communication (*p*=0.0108), disrespectful office staff (*p*=0.0062), and discriminatory behaviors (*p*=0.0261) in a clinical setting.

**Table 3.** Associations Between Select Patient-Level Factors and Interpersonal Processes of Care Subdomains

	Trust in physician		CARE measure		Stereotype vulnerability		Everyday discrimination		Self-reported health	
Unit change	5		10		1		5		1	
	β	p	β	p	β	p	β	p	β	p
IPC Domain 1: Hurried communication (−)	−0.35308	0.0283	−0.38081	<0.0001	−0.1174	0.2738	0.18438	0.0108	0.00829	0.9682
IPC Domain 2: Elicited concerns, responded (+)	0.37644	0.041	0.40670	<0.0001	0.05207	0.664	−0.1511	0.0657	0.42775	0.0581
IPC Domain 3: Explained results, medications (+)	0.91319	0.0004	0.52378	0.0003	0.12101	0.491	−0.2441	0.0485	0.37281	0.2673
IPC Domain 4: Patient-centered decision making (+)	0.98881	0.0001	0.59108	<0.0001	0.08647	0.6332	−0.2894	0.0192	0.30478	0.368
IPC Domain 5: Compassionate, respectful (+)	0.71995	0.0019	0.55767	<0.0001	0.30411	0.047	−0.1782	0.1005	0.29911	0.3058
IPC Domain 6: Discriminated (−)	−0.39985	0.1458	−0.40498	0.0052	0.05079	0.7735	0.26537	0.0261	0.10053	0.7613
IPC Domain 7: Disrespectful office staff (−)	−0.03982	0.8708	−0.37465	0.0031	0.02021	0.8963	0.27742	0.0062	0.33421	0.2463

Estimates were adjusted for age, sex, race, ethnicity, and education level. (−) indicates that a higher score=worse IPC; (+) indicates that a higher score=better IPC. Estimated coefficients (β) represent the estimated change in IPC for unit change in predictor, that is, a 5-unit change in the Trust in Physicians measure was associated with a 0.98881 increase in IPC Domain 4: Patient-centered decision making.

Trust and empathy were most consistently associated with the IPC, associated with five of seven and seven of seven subdomains, respectively. Stereotype vulnerability was significantly associated only with the perception that doctors demonstrated compassionate, respectful care (*p*<0.05). There was no significant association between any IPC subdomain and self-reported health. The race interaction term was not significant in any of the models, indicating that race differences in these relationships were not apparent in our study population.

### Correlations between patient-level factors

Among the three patient-level factors that were significantly associated with more than one IPC subdomain (i.e., empathy, trust, and discrimination), perceived empathy was found to be significantly correlated with trust in physicians (*r*=0.42, *p*=0.002) and perceived discrimination (*r*=−0.39, *p*=0.007). There was no significant correlation between trust in physicians and perceived discrimination (*r*=−0.13, *p*=0.40).

## Discussion

This study explored the association between five patient-level factors (trust, empathy, stereotype threat, everyday discrimination, and self-reported health) and patient perceptions of care, as measured by IPC subdomain scores. We set out to elucidate potential mechanisms for racial health care disparities to inform future intervention design. In our small sample, we did not detect significant race interactions in the relationship between the IPC subdomains and patient-level factors. While some data suggest that the relationship between key patient factors and perception of care is consistent across racial/ethnic groups, other studies suggest otherwise. For example, there is strong evidence that black patients have lower levels of trust in their physician^36,37^ and in the health system.^38^

**FIG. 1.**
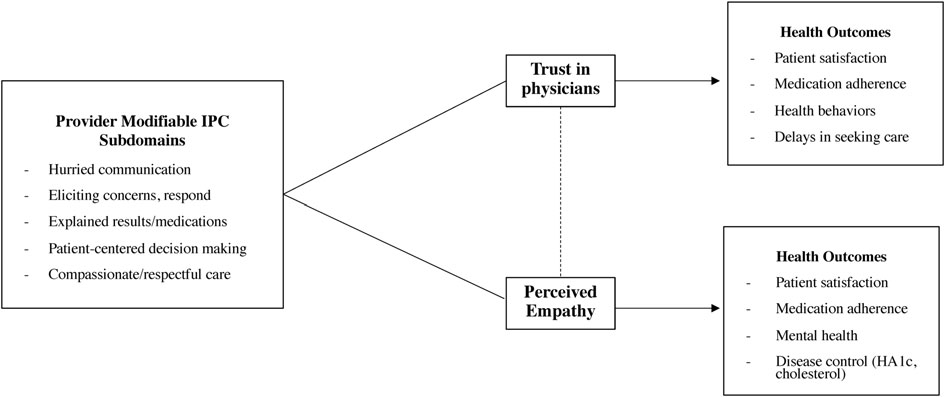
Conceptual model representing the relationship between Trust, Empathy, and IPC subdomains. *Dotted line* indicates correlation; could potentially include *r* and *p*-value. IPC, Interpersonal Processes of Care.

Overall, while not answering questions about racial differences, these results provide preliminary evidence that increased trust in physicians and perceived empathy are associated with positive perception of care, and increased perceived everyday discrimination is associated with negative perception of care. These findings are notable because, based upon the IPC, they suggest provider behaviors that are amendable to intervention through education and training. Shown in Figure 1, we developed a conceptual model for the relationship between trust, empathy, and provider modifiable IPC subdomains. The right side of the model, derived from previous literature, depicts the link between trust, empathy, and health outcomes. It is important to note that we were unable to determine directionality within the scope of this study, that is, whether perception of quality care affects trust and empathy perceived in the clinical setting or pre-existing trust and perceived empathy affect how a patient perceives the quality of their care. Regardless of directionality, these results present modifiable factors that may potentially improve downstream health outcomes.

Our data highlight the potential importance of empathy in the clinical encounter; perception of empathy was significantly associated with all subdomains of the IPC. Others have reported that the perception of physician empathy is associated with increased patient satisfaction and adherence, improved health outcomes, and reduced patient anxiety.^39–41^ Educational interventions can be effective in improving empathy and combating empathy reduction during medical school,^42^ and it is likely that providers can learn to effectively communicate the empathy and compassion they naturally feel for their patients.

Notably, a 5-unit increase in a measure of the patient's trust in their provider was strongly and positively associated with ∼1-unit increase in the communication-related IPC subdomains of patient-centered decision making and explaining results and medications. These results are consistent with previous literature demonstrating a positive association between patient trust and perception of care quality.^43,44^ This study builds upon this literature by suggesting that patient's trust in the provider, an important predictor of treatment adherence, may be increased by intervention designed to increase effective provider communication.^45–49^

Perceived everyday discrimination is a psychosocial stressor linked to negative psychological and physical health outcomes. It is associated with poor mental health,^50^ serving as an independent predictor of depressive symptoms, smoking, and substance use.^51–53^ Everyday discrimination has also been found to be associated with physical health, including hypertension,^54,55^ obesity,^56^ and markers of inflammation.^57,58^ Unlike measures for trust and empathy, which focus on the clinical context, perceived everyday discrimination is a measure of patients' experiences in society. Its association to the IPC is noteworthy as it provides better understanding of what may lead to the perception of discrimination and suggests ways to potentially mitigate it in the clinical encounter.

The observed lack of association between self-reported health and perception of care warrants further exploration. We found no association in our sample potentially due to our generally healthy patient pool, with a median health status of 4 (very good) and no participant identifying with “poor” health. Analysis of a sicker population may yield different results. It is possible that patients who are healthier attribute some of their health to the care they have received. Alternatively, those who perceive that their care has been patient centered may be more likely to also perceive that their overall health is good. One's perception of health status is an independent predictor of mortality.^59,60^ Therefore, a relationship between that outcome and potentially mutable characteristics of the care experience may suggest additional targets for intervention.

An unexpected finding was the general lack of relationship between IPC subdomains and stereotype vulnerability. Perception of compassionate, respectful care was the only subdomain of the IPC significantly associated with stereotype vulnerability. There are few studies linking stereotype vulnerability and health disparities^32,61^; however, it is speculated that stereotype threat in the clinical setting may contribute to impaired communication between patients and providers, avoidance of health care, and reduced adherence to treatment plan.^25,62^ A potential explanation for the observed lack of association is that the scale we used was originally developed in the context of academic test performance; questions are not health care related and it may be difficult for patients to extrapolate to a clinical context. Patients may also be less aware of group stereotypes in the health care setting. In addition, stereotype vulnerability may be more significant in patients from highly stigmatized groups (e.g., sickle cell and HIV) that were not included in our sample. Further research is needed to examine the impact of stereotype vulnerability in the health care setting.

As noted above, in our small sample, the relationship between the IPC subdomains and patient-level factors did not differ by race. While this was an unexpected finding, it is consistent with current literature using the IPC. In stratified analyses, Nápoles et al. found that several IPC subdomains (compassionate/respectful care, eliciting/responding to patient concerns, and patient-centered decision making) were positively and strongly associated with patient satisfaction in English- and Spanish-speaking Latinx African Americans, and non-Latinx whites.^63^ While some data suggest that the relationship between key patient factors and perception of care is consistent across racial/ethnic groups, other studies suggest otherwise. For example, there is strong evidence that black patients have lower levels of trust in their physician^64,65^ and in the health system.^66^ Although we did not have sufficient power to evaluate racial and ethnic differences, these differences (or lack thereof) warrant additional study.

### Correlations between patient-level factors

To determine if the patient-level factors were surrogates for each other, we determined correlations between them. Although we found that empathy is related to both trust and discrimination, the weak correlation among these factors suggests that they can be considered and analyzed independently. The lack of correlation between trust in physician and perceived everyday discrimination may reflect the core importance to patients of trust in the patient-physician relationship, and tendency of patients to have high levels of trust in their own medical providers.^65,66^ This finding may also be a result of the measure itself—as noted above, the Everyday Discrimination Scale is not specific to the health care setting. In addition, findings may have differed if we had assessed trust in the health care system in general.^66^

### Limitations

The major limitations of this study include small sample size, limited generalizability, and potential recall bias. Although the focus group study that determined our study population was deliberately diverse by race, ethnicity, and socioeconomic status, there was limited power to detect potential differences by race or ethnicity. In addition, the study population was not fully representative: the participant pool was younger, more educated, and more female than the general population. The pool also did not include patients with low English proficiency, preventing analysis of language as a potential mediator of perception. There was limited representation of medical conditions and levels of morbidity; clearly assessing the clinical experience in sicker patients is important. Future research will benefit from a larger and more representative study population. In addition, due to survey-based methods and inclusion criteria of at least two nonurgent ambulatory care visits within the last 12 months, recall bias could have played a role in the results obtained. Survey responses may be skewed depending on number, type, and timing of visits.

## Conclusion

This exploratory study suggests that patient perception of quality care is significantly linked to empathy, trust, and perceived everyday discrimination. Results present modifiable factors that may potentially improve patient perception and downstream health outcomes. Future research should focus on further characterizing factors associated with the IPC and determining the impact on health outcomes of interventions designed to improve processes of care. In addition, future research should shed additional light on potential race differences in these relationships.

### Health equity implications

The IPC provides modifiable factors for providers to potentially improve patient perceptions of care and consequentially the patient care experience. Each IPC subdomain (hurried communication, eliciting and responding to concerns, explaining results and medications, etc.) is an actionable item that may be incorporated into future interventions and quality improvement efforts centering around the clinical encounter. Focus on improving perceptions may be important in improving health outcomes and efficiency of care. As health care shifts toward patient centeredness, patient perception has also become an important metric in the evaluation of quality of care.^5,6,66^ These preliminary data suggest that increased efforts to improve clinician communication of empathy and general communication skill may have a positive effect on this perception.

## Supplementary Material

Supplemental data

## Supplementary Material

Supplementary Appendix SA1

## Abbreviations Used

CARE: consultation and relational empathy
IPC: Interpersonal Processes of Care
REACH Equity: Research to Advance Healthcare Equity
SD: standard deviation
SVS: Stereotype Vulnerability Scale
