# Supplementary material for: The Patient's Point of View: Characterizing Patient-Level Factors Associated with Perceptions of Health Care
Source: Health Equity. 2021 Jun 25;5(1):457–65. doi: 10.1089/heq.2021.0062 (PMC8252902; doi:10.1089/heq.2021.0062)
Supplement: Supplemental data [file Supp_AppendixSA1.docx]

# Interpersonal Processes of Care Survey

**Range:** 1-5

**Description:** 7 subscales, where meaning/direction of score varies depending upon the scale. The IPC survey can be used to describe disparities in interpersonal care, predict patient outcomes, and examine outcomes of quality improvement efforts to reduce health care disparities.

**Reference:** Stewart AL, Nápoles-Springer A, Gregorich SE and Santoyo-Olsson J. Interpersonal processes of care: Patient-reported measures for diverse groups. *Health Serv Res*, 2007; 42 (3, Part I):1235-1256. PMID: 17489912

**Communication:** The following questions are about your experiences talking with your doctors at (name of site) over the past 12 months.

## Hurried communication

1. How often did doctors speak too fast? (SF)
2. How often did doctors use words that were hard to understand? (SF)
3. How often did doctors ignore what you told them?
4. How often did doctors appear to be distracted when they were with you?
5. How often did doctors seem bothered if you asked several questions?

## Elicited concerns, responded

1. How often did doctors really find out what your concerns were? (SF)
2. How often did doctors let you say what you thought was important? (SF)
3. How often did doctors take your health concerns very seriously? (SF)

## Explained results, medications

1. How often did doctors explain your test results such as blood tests, X-rays, or cancer screening tests? (SF)
2. How often did doctors clearly explain the results of your physical exam? (SF)
3. How often did doctors tell you what could happen if you didn’t take a medicine that they

prescribed for you?

1. How often did doctors tell you about side effects you might get from a medicine?

**Decision making:** These questions are about how you and your medical doctors decide about your health care. Please continue to think about your experiences over the past 12 months.

## Patient-centered decision making

1. How often did doctors ask if you would have any problems following what they recommended?
2. How often did doctors ask if you felt you could do the recommended treatment?
3. How often did you and your doctors work out a treatment plan together? (SF)
4. If there were treatment choices, how often did doctors ask if you would like to help decide your

treatment? (SF)

Interpersonal style: The following questions are about the personal interactions between you and your doctors over the past 12 months.

## Compassionate, respectful

1. How often were doctors compassionate?
2. How often did doctors give you support and encouragement?
3. How often were doctors concerned about your feelings? (SF)
4. How often did doctors really respect you as a person? (SF)
5. How often did doctors treat you as an equal? (SF)

## Discrimination

1. How often did doctors make assumptions about your level of education?
2. How often did doctors make assumptions about your income?
3. How often did doctors pay less attention to you because of your race or ethnicity? (SF)
4. How often did you feel discriminated against by doctors because of your race or ethnicity? (SF)

## Disrespectful office staff

1. How often was office staff rude to you? (SF)
2. How often did office staff talk down to you? (SF)
3. How often did office staff give you a hard time? (SF)
4. How often did office staff have a negative attitude toward you? (SF)

Note: Response choices: 1, never; 2, rarely; 3, sometimes; 4, usually; 5, always.

** SF indicates that item is part of the 18-item short form.

# Trust in Physician Scale

**Range:** 5-25

**Description:** Brief scale to measure trust in physician, where higher scores indicate more trust

**Reference:** Dugan, Elizabeth, Felicia Trachtenberg, and Mark A. Hall. "Development of abbreviated measures to assess patient trust in a physician, a health insurer, and the medical profession." *BMC health services research* 5.1 (2005): 64.

Responses are strongly disagree to strongly agree

- 1. I completely trust this doctor’s decisions about which treatments are best for me [or my family member]
  2. This doctor is extremely thorough and careful
  3. Sometimes this doctor cares more about what is convenient for (him/her) than about my medical needs [my family member’s

medical needs]

- 1. This doctor is totally honest with me
  2. All in all, I have complete trust in this doctor

# Consultation and Relational Empathy (CARE) Measure

**Range:** 10-50, with higher scores indicating greater perceptions of empathy

**Description:** Tool measuring patients’ perceptions of relational empathy in clinical settings

## References:

Mercer, S.W. and Reynolds, W. (2002), “Empathy and quality of care”, British Journal of General Practice, Vol. 52, supplement, pp. S9-S12.

Mercer, S. W., Maxwell, M., Heaney, D., & Watt, G. (2004). The consultation and relational empathy (CARE) measure: development and preliminary validation and reliability of an empathy-based consultation process measure. Family practice, 21(6), 699-705.

| **Thinking about your primary doctor, how was the doctor at:** | | | | | | |
| --- | --- | --- | --- | --- | --- | --- |
|  | Poor 1 | Fair 2 | Good 3 | Very Good  4 | Excellent 5 | Does Not  Apply |
| **1. Making you feel at ease……** *(being friendly and warm towards you, treating you with respect,*  *not cold or abrupt)* | □ | □ | □ | □ | □ | □ |
| **2. Letting you tell your “story”**  **…...**  *(giving you time to fully describe your illness in your own words;*  *not interrupting or diverting you)* | □ | □ | □ | □ | □ | □ |
| **3. Really listening……**  *(paying close attention to what you were saying not looking at the notes or computer when you*  *were talking)* | □ | □ | □ | □ | □ | □ |
| **4. Being interested in you as a**  **whole person……** | □ | □ | □ | □ | □ | □ |

| *(asking/knowing relevant details about your life, your situation; not treating you as “just a number”)* |  |  |  |  |  |  |
| --- | --- | --- | --- | --- | --- | --- |
| **5. Fully understanding your**  **concerns…...**  *(communicating that he/she had accurately understood your*  *concerns not overlooking or dismissing anything)* | □ | □ | □ | □ | □ | □ |
| **6. Showing care and**  **compassion……**  *(seemingly genuinely concerned, connecting with you on a human level; not being indifferent of*  *“detached”)* | □ | □ | □ | □ | □ | □ |
| **7. Being positive……**  *(having a positive approach and a positive attitude; being honest but not negative about your*  *problems)* | □ | □ | □ | □ | □ | □ |
| **8. Explaining things clearly……** *(fully answering your questions, explaining clearly, giving you adequate information; not being*  *vague)* | □ | □ | □ | □ | □ | □ |
| **9. Helping you take control……** *(exploring with you what you can do to improve your health yourself; encouraging rather than*  *“lecturing” you)* | □ | □ | □ | □ | □ | □ |
| **10. Making a plan of action with**  **you……**  *(discussing the options, involving you in decisions as much as you want to be involved; not ignoring*  *your views)* | □ | □ | □ | □ | □ | □ |

# Revised Stereotype Vulnerability Scale (SVS-4)

**Range:** 1-5, with higher scores indicating greater vulnerability

**Description:** Revised version of the Stereotype Vulnerability Scale originally developed to assess stereotype threat in women’s math

performance

## References:

Woodcock, A., Hernandez, P. R., Estrada, M., & Schultz, P. (2012). The consequences of chronic stereotype threat:Domain disidentification and abandonment. Journal of personality and social psychology, 103 (4), 635.

Spencer, S. J., Steele, C. M., & Quinn, D. M. (1999). Stereotype threat and women's math performance. Journal of experimental social psychology , 35 (1), 4-28.

## Because of your ethnicity:

|  | Almost  Always 5 | Often 4 | Sometimes 3 | Seldom 2 | Never 1 |
| --- | --- | --- | --- | --- | --- |
| **1. Some people believe that you have less ability.** | □ | □ | □ | □ | □ |
| **2. If you’re not better than average, people assume you are limited.** | □ | □ | □ | □ | □ |
| **3. If you do poorly on a test, people will assume that it is because of your ethnicity.** | □ | □ | □ | □ | □ |
| **4. People of your ethnicity face unfair evaluations because of their ethnicity.** | □ | □ | □ | □ | □ |

Everyday Discrimination Scale

**Range:** 0-45, with higher scores indicating greater perceived discrimination **Description:** A widely used measure of perceived discrimination **References:**

Williams, D.R., Yu, Y., Jackson, J.S., and Anderson, N.B. “Racial Differences in Physical and Mental Health: Socioeconomic Status,

Stress, and Discrimination.” Journal of Health Psychology. 1997; 2(3):335-351.

Krieger N., Smith K., Naishadham D., Hartman C., Barbeau E.M. “Experiences of

discrimination: validity and reliability of a self-report measure for population health

research on racism and health.” Social Science & Medicine. 2005; 61(7):1576-1596. o Taylor T.R.,

Kamarck T.W., Shiffman S. “Validation of the Detroit area study discrimination scale in a community sample of older African

American adults: the Pittsburgh healthy heart project.” International Journal of Behavioral Medicine. 2004; 11:88–94.

## In your day-to-day life, how often do any of the following things happen to you?

| **1. You are treated with less courtesy than other people are.** | | | | | | | | | | | | | | | | | |
| --- | --- | --- | --- | --- | --- | --- | --- | --- | --- | --- | --- | --- | --- | --- | --- | --- | --- |
| Almost Everyday | | | At least once a week | | | A few times a month | | A few times a year | | | Less than once a year | | | | Never | | |
| **If a few times a year or more, what do you think is the main reason for these experiences? (circle all that apply)** | | | | | | | | | | | | | | | | | |
| Ancestry/National Origins | | Gender | | Race | Age | | Religion | Height | | Weight | | Some other aspect of  physical appearance | | Sexual Orientation | | | Education of Income Level |
| **2. You are treated with less respect than other people are.** | | | | | | | | | | | | | | | | | |
| Almost Everyday | At least once a week | | | | A few times a month | | | | A few times a year | | | | Less than once a year | | | Never | |
| **If a few times a year or more, what do you think is the main reason for these experiences? (circle all that apply)** | | | | | | | | | | | | | | | | | |
| Ancestry/National Origins | | Gender | | Race | Age | | Religion | Height | | Weight | | Some other aspect of physical  appearance | | Sexual Orientation | | | Education of Income Level |
| **3. You receive poorer service than other people at restaurants or stores.** | | | | | | | | | | | | | | | | | |

| Almost Everyday | At least once a week | | | A few times a month | | | A few times a year | | | Less than once a year | | Never | |
| --- | --- | --- | --- | --- | --- | --- | --- | --- | --- | --- | --- | --- | --- |
| **If a few times a year or more, what do you think is the main reason for these experiences? (circle all that apply)** | | | | | | | | | | | | | |
| Ancestry/National | | Gender | Race | Age | Religion | Height | | Weight | Some other | | Sexual | | Education of |
| Origins | |  |  |  |  |  | |  | aspect of | | Orientation | | Income Level |
|  | |  |  |  |  |  | |  | physical | |  | |  |
|  | |  |  |  |  |  | |  | appearance | |  | |  |
| **4. People act as if they think you are not smart.** | | | | | | | | | | | | | |
| Almost Everyday | At least once a week | | | A few times a month | | | A few times a year | | | Less than once a year | | Never | |
| **If a few times a year or more, what do you think is the main reason for these experiences? (circle all that apply)** | | | | | | | | | | | | | |
| Ancestry/National | | Gender | Race | Age | Religion | Height | | Weight | Some other | | Sexual | | Education of |
| Origins | |  |  |  |  |  | |  | aspect of | | Orientation | | Income Level |
|  | |  |  |  |  |  | |  | physical | |  | |  |
|  | |  |  |  |  |  | |  | appearance | |  | |  |

**5. People act as if they are afraid of you.**

| Almost Everyday | | | At least once a week | | | A few times a month | | A few times a year | | | Less than once a year | | | | Never | | |
| --- | --- | --- | --- | --- | --- | --- | --- | --- | --- | --- | --- | --- | --- | --- | --- | --- | --- |
| **If a few times a year or more, what do you think is the main reason for these experiences? (circle all that apply)** | | | | | | | | | | | | | | | | | |
| Ancestry/National Origins | | Gender | | Race | Age | | Religion | Height | | Weight | | Some other aspect of physical  appearance | | Sexual Orientation | | | Education of Income Level |
| **6. People act as if they think you are dishonest.** | | | | | | | | | | | | | | | | | |
| Almost Everyday | At least once a week | | | | A few times a month | | | | A few times a year | | | | Less than once a year | | | Never | |
| **If a few times a year or more, what do you think is the main reason for these experiences? (circle all that apply)** | | | | | | | | | | | | | | | | | |
| Ancestry/National Origins | | Gender | | Race | Age | | Religion | Height | | Weight | | Some other aspect of physical  appearance | | Sexual Orientation | | | Education of Income Level |
| **7. People act as if they’re better than you are.** | | | | | | | | | | | | | | | | | |

| Almost Everyday | At least once a week | | | A few times a month | | | A few times a year | | | Less than once a year | | Never | |
| --- | --- | --- | --- | --- | --- | --- | --- | --- | --- | --- | --- | --- | --- |
| **If a few times a year or more, what do you think is the main reason for these experiences? (circle all that apply)** | | | | | | | | | | | | | |
| Ancestry/National | | Gender | Race | Age | Religion | Height | | Weight | Some other | | Sexual | | Education of |
| Origins | |  |  |  |  |  | |  | aspect of | | Orientation | | Income Level |
|  | |  |  |  |  |  | |  | physical | |  | |  |
|  | |  |  |  |  |  | |  | appearance | |  | |  |
| **8. You are called names or insulted.** | | | | | | | | | | | | | |
| Almost Everyday | At least once a week | | | A few times a month | | | A few times a year | | | Less than once a year | | Never | |
| **If a few times a year or more, what do you think is the main reason for these experiences? (circle all that apply)** | | | | | | | | | | | | | |
| Ancestry/National | | Gender | Race | Age | Religion | Height | | Weight | Some other | | Sexual | | Education of |
| Origins | |  |  |  |  |  | |  | aspect of | | Orientation | | Income Level |
|  | |  |  |  |  |  | |  | physical | |  | |  |
|  | |  |  |  |  |  | |  | appearance | |  | |  |
| **9. You are threatened or harassed.** | | | | | | | | | | | | | |
| Almost Everyday | At least once a week | | | A few times a month | | | A few times a year | | | Less than once a year | | Never | |
| **If a few times a year or more, what do you think is the main reason for these experiences? (circle all that apply)** | | | | | | | | | | | | | |
| Ancestry/National | | Gender | Race | Age | Religion | Height | | Weight | Some other | | Sexual | | Education of |
| Origins | |  |  |  |  |  | |  | aspect of | | Orientation | | Income Level |
|  | |  |  |  |  |  | |  | physical | |  | |  |
|  | |  |  |  |  |  | |  | appearance | |  | |  |

Self-rated Health

**Range:** 1-5 with higher score indicating better health.

**Description:** A single-item, self-report indicator of general health status.

## Reference:

Ware JE, Davies-Avery A, Donald C. Conceptualisation and measurement of health for adults in the health insurance study. Vol V. General health perceptions. Santa Monica, CA: Rand, 1978;

Stewart AL, Ware JE, eds. Measuring functioning and well-being. The medical outcomes study approach. Durham: Duke University Press, 1992.

## In general, would you say that your health is:

| Excellent 5 | Very Good 4 | Good 3 | Fair 2 | Poor 1 |
| --- | --- | --- | --- | --- |
| □ | □ | □ | □ | □ |
